# Topical Antibiotic Use Coselects for the Carriage of Mobile Genetic Elements Conferring Resistance to Unrelated Antimicrobials in Staphylococcus aureus

**DOI:** 10.1128/AAC.02000-17

**Published:** 2018-01-25

**Authors:** Glen P. Carter, Mark B. Schultz, Sarah L. Baines, Anders Gonçalves da Silva, Helen Heffernan, Audrey Tiong, Peter H. Pham, Ian R. Monk, Timothy P. Stinear, Benjamin P. Howden, Deborah A. Williamson

**Affiliations:** a Department of Microbiology & Immunology, Doherty Institute, The University of Melbourne, Melbourne, Australia; b Doherty Applied Microbial Genomics, Doherty Institute, The University of Melbourne, Melbourne, Australia; c Microbiological Diagnostic Unit Public Health Laboratory, Department of Microbiology & Immunology, Doherty Institute, The University of Melbourne, Melbourne, Australia; d Institute of Environmental Science and Research, Wellington, New Zealand

**Keywords:** fusidic acid, mupirocin, Staphylococcus aureus, topical antibiotics, coselection, multidrug resistance, plasmids

## Abstract

Topical antibiotics, such as mupirocin and fusidic acid, are commonly used in the prevention and treatment of skin infections, particularly those caused by staphylococci. However, the widespread use of these agents is associated with increased resistance to these agents, potentially limiting their efficacy. Of particular concern is the observation that resistance to topical antibiotics is often associated with multidrug resistance, suggesting that topical antibiotics may play a role in the emergence of multidrug-resistant (MDR) strains. New Zealand (NZ) has some of the highest globally recorded rates of topical antibiotic usage and resistance. Using a combination of Pacific Biosciences single-molecule real-time (SMRT) whole-genome sequencing, Illumina short-read sequencing, and Bayesian phylogenomic modeling on 118 new multilocus sequence type 1 (ST1) community Staphylococcus aureus isolates from New Zealand and 61 publically available international ST1 genome sequences, we demonstrate a strong correlation between the clinical introduction of topical antibiotics and the emergence of MDR ST1 S. aureus. We also provide *in vitro* experimental evidence showing that exposure to topical antibiotics can lead to the rapid selection of MDR S. aureus isolates carrying plasmids that confer resistance to multiple unrelated antibiotics, from within a mixed population of competitor strains. These findings have important implications regarding the impact of the indiscriminate use of topical antibiotics.

## INTRODUCTION

Topical antibiotics, such as mupirocin and fusidic acid (FA), are commonly used in the prevention and treatment of skin infections, particularly those caused by staphylococci (1). However, the widespread use of topical antimicrobials has been associated with increased bacterial resistance, limiting the potential efficacy of these agents (2–4). For example, in New Zealand (NZ), the unrestricted use of mupirocin throughout the 1990s resulted in high levels of mupirocin resistance (28%) among community isolates of Staphylococcus aureus (5). Similarly, a marked increase in the level of community dispensing rates for topical FA in NZ was associated with an increase in FA resistance from 17% in 1999 to 28% in 2015, representing one of the highest rates of S. aureus FA resistance globally (3). The prolonged high usage of topical antibiotics in NZ provides a valuable opportunity to study the temporal evolution of resistance to these agents across S. aureus lineages. Recently, we described the emergence of three dominant FA-resistant S. aureus clones in NZ: a multilocus sequence type 5 (ST5) methicillin-resistant S. aureus (MRSA) clone, an ST1 MRSA clone, and an ST1 methicillin-susceptible S. aureus (MSSA) clone (3). In each clone, FA resistance was mediated by the *fusC* gene, which was located within staphylococcal cassette chromosome (SCC) elements, with or without *mecA* (6). Of concern, we observed that approximately 50% of FA-resistant ST1 S. aureus strains were coresistant to mupirocin, another commonly used topical antibiotic (3).

Here, to further understand the impact of topical antibiotic use on the emergence of antimicrobial resistance, we performed a detailed genomic analysis of ST1 S. aureus. This lineage is dominant in the NZ community and is also globally prevalent (7–9). We demonstrate a strong correlation between the clinical introduction of topical antibiotics and the emergence of multiresistant ST1 S. aureus in NZ. Furthermore, we provide *in vitro* experimental evidence showing that exposure to topical antibiotics can rapidly select for isolates resistant to several unrelated classes of antimicrobials from within a mixed population of competitor strains. Our findings demonstrate the impact that the indiscriminate use of topical antibiotics can have on antimicrobial resistance rates within the community.

## RESULTS

### Topical antibiotic resistance genes are encoded on stable mobile genetic elements in ST1 S. aureus.

**FIG 1:**
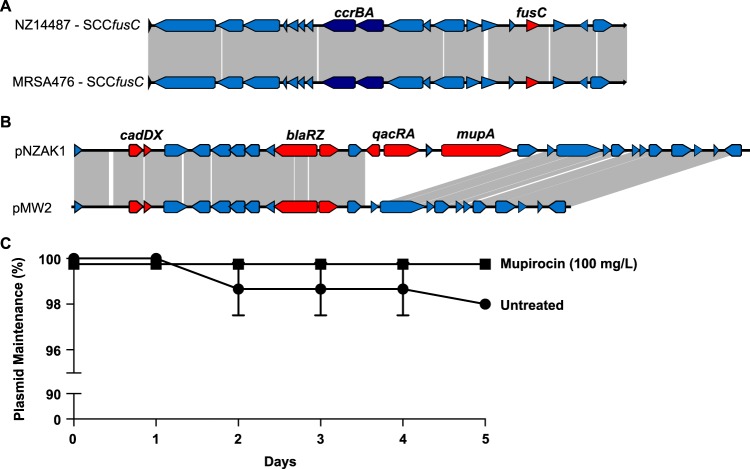
(A) Schematic diagram illustrating the genetic organization of the SCC*fusC* region of NZ14487 in comparison to the *fusC*-harboring SCC_476_ element of MRSA476 (GenBank accession no. NC_002953). Regions of similarity are joined by gray lines. The *fusC* and *ccrBA* genes are highlighted. (B) Schematic diagram depicting the linear genomic comparisons of plasmid pNZAK1 from S. aureus strain NZ14487 and plasmid pMW2 from S. aureus strain MW2 (GenBank accession no. AP004832). Regions of DNA similarity are joined by gray lines. The location of predicted antimicrobial resistance genes (*cadDX*, *blaRZ*, *qacRA*, and *mupA*) are shown with red arrows. (C) Segregational stability of plasmid pNZAK1 in Staphylococcus aureus NZ14487. Cultures of S. aureus NZ14487 were serially passaged in either nonselective BHI broth or in broth supplemented with 100 mg/liter mupirocin. Plasmid loss was determined at the days indicated. The mean of triplicate independent cultures grown under each test condition are shown, with error bars representing the standard error of the mean (SEM).

To define the genetic context of topical antimicrobial resistance determinants, a representative ST1 MSSA (NZ14487) genome sequence was generated using single-molecule real-time (SMRT) sequencing (Pacific Biosciences). The genome of NZ14487 comprised a circular chromosome of 2,846,702 bp (GC content, 34%) and a single circular plasmid (termed pNZAK1) of 27,938 bp (GC content, 33%). *In silico* multilocus sequence typing (MLST) confirmed that this isolate belonged to the ST1 lineage. The *fusC* gene was located within an ∼26-kb SCC element, with 99.9% nucleotide sequence identity to the *fusC*-harboring SCC_476_ element (GenBank accession no. NC_002953), previously described in ST1 MSSA strains from the United Kingdom and New Zealand (Fig. 1A) (6, 7, 10). The *mupA* gene (mediating high-level mupirocin resistance) was colocated downstream of the *qacA* gene on a resistance cassette on pNZAK1. The *qacA* gene encodes an efflux pump, QacA, which has previously been associated with biocide tolerance in S. aureus (11, 12). This antimicrobial resistance cassette was 7,285 nucleotides in length and appeared to be inserted between nucleotides 12154 and 12155 of plasmid pMW2 (accession no. NC_005011). In addition to *qacA* and *mupA*, the cassette also contained *qacR* upstream of *qacA*, a hypothetical protein-encoding gene in the intergenic region between *qacA* and *mupA*, and a fructosamine kinase gene downstream of *mupA*. There was no evidence of any transposase or resolvase genes within the region, and we were also unable to identify any direct repeats, inverted repeats, or tandem repeats flanking the region, which might indicate that the cassette was part of an independent mobile genetic element. We were also unable to find any IS*257* elements within the cassette, despite these insertion elements being frequently associated with both *mupA* and *qacA* (13, 14). There was, however, an ISEL*39* insertion element located in the intergenic region between *qacA* and *mupA*. The *mupA* and *qacA-qacR* cassette contained regions of homology to several different staphylococcal plasmids, including pAMT2 from Staphylococcus epidermidis strain ATCC 12228 (accession no. NZ_CP022250) and p14035 from S. aureus strain CC1 (accession no. KY465818), suggesting that this cassette may have arisen following one or more interplasmidic recombination events.

**FIG 2:**
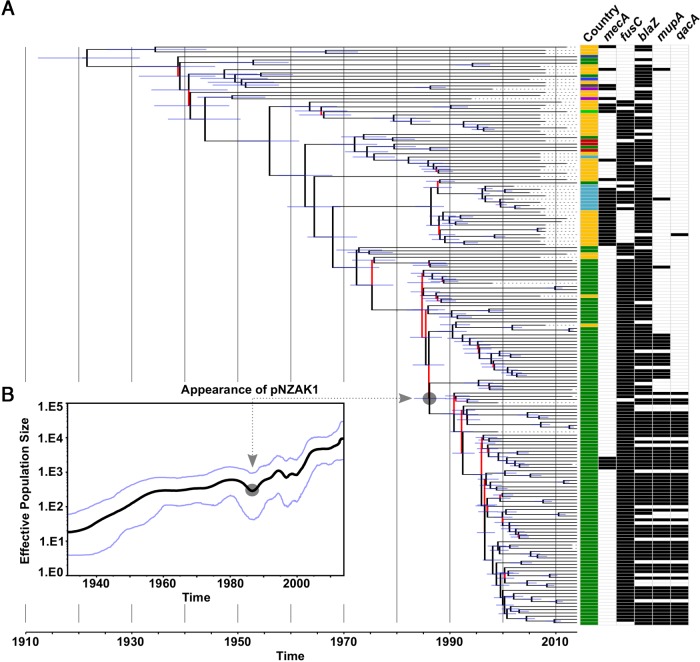
Evolutionary history of the S. aureus ST1 lineage in NZ. (A) Maximum clade credibility (MCC) tree, generated in BEAST version 1.8.3 from 14,350-site filtered single-nucleotide polymorphisms (SNPs) in the core genome of 179 isolates, illustrating the global phylogeny of ST1 S. aureus. Branches are colored to reflect statistical support; those possessing a posterior probability of <95% are red. The 95% highest posterior density interval (HPDI) is shown by the blue horizontal lines. Branch annotations provide country of isolation (dark green, NZ; yellow, Australia; light blue, UK; red, Malaysia; light green, India; dark blue, Brazil; gray, Iraq, royal blue, France; purple, USA) and the *mecA*, *fusC*, *blaZ*, *mupA*, and *qacA* resistance gene status. The predicted pNZAK1 acquisition event is indicated. (B) Bayesian Skyline plot showing the change in the effective population size (EPS) of the ST1 lineage with time. The median EPS is indicated by the central black line, and the 95% HPDI is bordered by the blue lines above and below the median value. As described for panel A, the predicted pNZAK1 acquisition event is indicated.

Notably, except for the *mupA-qacA*-containing region, pNZAK1 displayed a high level of similarity to pMW2 (GenBank accession no. AP004832) (Fig. 1B), a common plasmid among diverse community S. aureus isolates (15). To investigate the stability of pNZAK1, we performed segregational stability assays with NZ14487 under selective and nonselective conditions. pNZAK1 was highly stable, with a segregational stability of 98% after 5 days of serial passage under nonselective growth conditions (Fig. 2A). Although not significantly different, the segregational stability of pNZAK1 increased to 100% in growth medium supplemented with 100 mg/liter mupirocin (Fig. 1C).

### Topical antimicrobial use is temporally associated with the emergence of distinct ST1 lineages.

To investigate the evolutionary history of the ST1 lineage in NZ, phylogenetic reconstruction was performed using BEAST (28) (Fig. 2A). Whole-genome sequencing (WGS) was performed on 118 NZ ST1 isolates, and the collection was supplemented with sequence reads from 61 international ST1 isolates (Table S1 in the supplemental material). Bayesian analysis estimated the median rate of nucleotide substitutions to be 1.38 × 10^−6^ mutations per site per year (95% highest posterior density interval [HPDI], 1.25 × 10^−6^ to 1.52 × 10^−6^ mutations per site per year), largely consistent with the 95% HPDI obtained for other S. aureus strains (16). Our analysis predicted that the most recent common ancestor (MRCA) of all sampled ST1 isolates arose in 1922 (95% HPDI, 1912 to 1933) and that the effective population size (EPS) of the ST1 lineage underwent two major expansions (Fig. 2B). The first bacterial population increase was between the 1930s and the late 1950s, followed by a relative plateau until the early 1990s. A dramatic increase in the EPS of ST1 S. aureus was observed from the late 1990s and has not yet reached an obvious plateau.

Next, we investigated the temporal emergence of specific antimicrobial resistance (AMR) genes (*blaZ*, *fusC*, *mupA*, and *qacA*) across the evolutionary history of the ST1 lineage by mapping the presence of these genes onto the phylogeny (Fig. 2A). The year of acquisition of *blaZ* into this population was approximately 1922 (95% HPDI, 1912 to 1933). The acquisition of *fusC* was more recent, with *fusC*-containing isolates emerging around 1956 (95% HPDI, 1950 to 1962). Isolates containing both *mupA* and *qacA* emerged concurrently in ∼1991 (95% HPDI, 1988 to 1993), likely representing a single acquisition of the *mupA*- and *qacA*-carrying plasmid pNZAK1. A second smaller group of isolates encoding *mupA* alone (i.e., not associated with *qacA*) arose separately in 1993 (95% HPDI, 1989 to 1995) following the acquisition of a *mupA*-containing plasmid unrelated to pNZAK1.

### Fusidic acid and mupirocin use can drive the emergence of MDR S. aureus.

**FIG 3:**
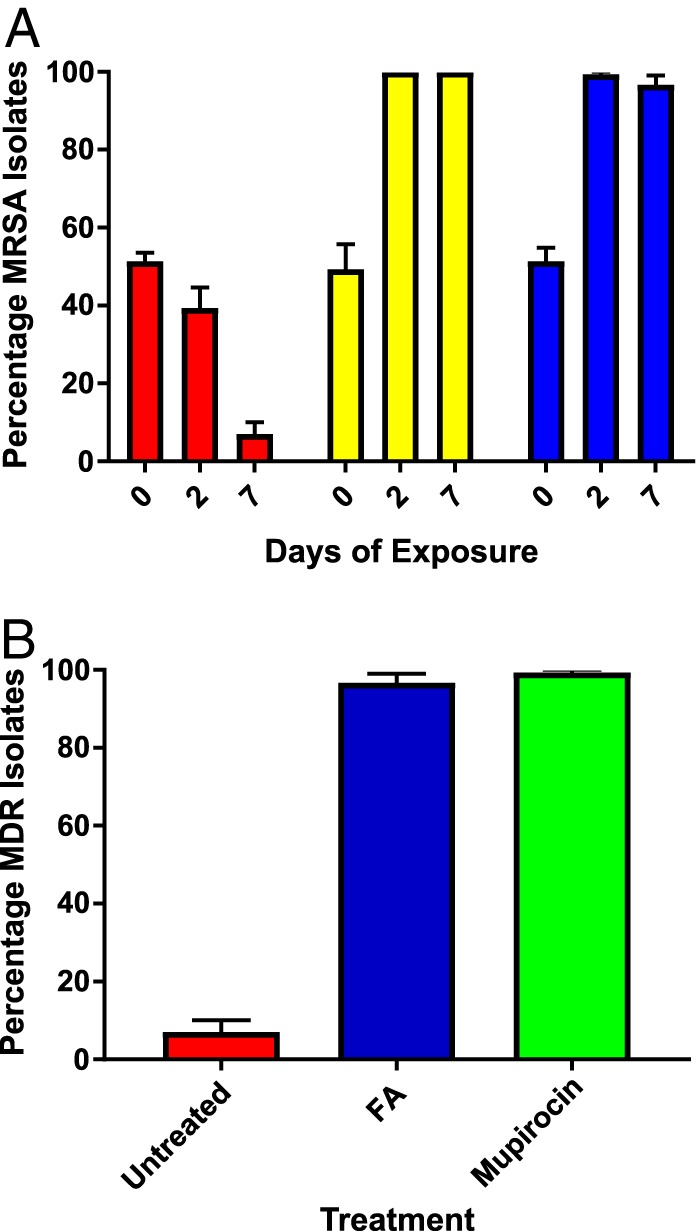
(A) Competitive index assays showing the proportion of MRSA within a mixed population of competing S. aureus isolates following nonselective growth (red bars), or following exposure to either 6 mg/liter oxacillin (yellow bars) or 8 mg/liter FA (blue bars) for the number of days indicated. (B) The proportion of MDR isolates within a mixed population of competing S. aureus strains following 7 days of nonselective growth (red) or 7 days of exposure to 8 mg/liter FA (blue) or 100 mg/liter mupirocin (green). For both panels, the data represent the mean of the results of three independent assays, with error bars showing the SEM.

Given the colocation of *fusC* in the same mobile SCC element as *mecA* in the MRSA isolates from this lineage, we hypothesized that FA exposure might drive the selection of MRSA. To explore this, we performed pairwise competition experiments using three different FA-resistant, pNZAK1-positive ST1 MRSA clinical isolates from NZ, paired with three FA-susceptible, pNZAK1-negative clinical MSSA isolates. Each pair was grown (i) under nonselective conditions or (ii) in growth medium supplemented with either 8 mg/liter FA or 6 mg/liter oxacillin. The ratio of MSSA to MRSA was then monitored to determine the resulting population structure. In these experiments, nonselective conditions favored the emergence of FA-susceptible MSSA isolates over FA-resistant MRSA isolates, with 61% and 93% of the isolates being MSSA after 2 and 7 days, respectively (Fig. 3A). As expected, oxacillin exposure resulted in the emergence of MRSA, with 100% of isolates being MRSA after both 2 and 7 days, respectively. Similarly, FA exposure also resulted in the dominance of MRSA, with 99% and 97% of isolates being MRSA after 2 and 7 days, respectively (Fig. 3A). Notably, under the conditions tested, there was no significant difference in the ability of FA or oxacillin to enrich for MRSA (*P* = 0.786), with exposure to either agent being significantly more likely to result in the emergence of MRSA isolates than under nonselective conditions (*P* < 0.0001 for both FA and oxacillin). Under these experimental conditions, FA also enriched for isolates that carried plasmid pNZAK1 and therefore displayed high-level mupirocin resistance (encoded by *mupA* on pNZAK1). After 7 days, only 7% of the population was MDR (i.e., resistant to FA, mupirocin, and oxacillin) following nonselective growth, compared to 97% of isolates following exposure to FA (Fig. 3B). WGS of representative MDR isolates confirmed that the resulting isolates were identical to the input strains and, importantly, contained the same antimicrobial resistance genes.

Given our observation that FA selected for an MDR clone *in vitro*, we hypothesized that mupirocin exposure may also select for MDR isolates of this lineage. Pairwise competition assays were therefore performed using the same three MRSA-MSSA pairings described above for FA exposure. Similar to FA, exposure to 100 mg/liter mupirocin resulted in 99% of isolates being MDR (resistant to FA, mupirocin, and penicillin) at the conclusion of the experiment (day 7) (Fig. 3B).

## DISCUSSION

In this study, we combined phylogenomic and phenotypic analyses to highlight the potential for topical antimicrobial usage to shape the population structure of S. aureus, focusing on the emergence of a prevalent S. aureus lineage in a country where topical antibiotic use has been largely unregulated for 2 decades. Our evolutionary modeling demonstrates an association between the clinical use of specific antibiotics and the emergence of distinct ST1 clades. For example, the initial expansion of *blaZ*-harboring ST1 isolates correlates temporally with mass clinical usage of penicillin from the late 1940s. Similarly, the emergence of mupirocin-resistant ST1 isolates (harboring pNZAK1) in the early 1990s is consistent with the introduction of mupirocin into clinical usage in NZ in 1986 (17). Between 1991 and 2000, mupirocin (Bactroban) was available to purchase over-the-counter in NZ, and high rates of use led to a marked increase in mupirocin resistance in S. aureus by the late 1990s (5). The latest expansion of *fusC*-harboring ST1 in NZ from the late 1990s onwards is contemporaneous with an increased usage of topical FA and reduced use of mupirocin. Previous work showed that topical FA use increased dramatically in NZ throughout the 2000s, and that the dominant MSSA and MRSA clones in NZ (ST1 MSSA and ST5 MRSA, respectively) are now both FA resistant (3). Our evolutionary modeling demonstrates the ongoing expansion of the FA-resistant ST1 population in NZ, likely driven by continuing high population usage of topical FA. Based on our analyses, it seems probable that unless the use of topical FA is restricted, resistance rates will continue to rise.

Our phenotypic analyses also demonstrate the potential for topical antibiotics to coselect for multiresistant isolates. For example, under the experimental conditions used here, we observed that FA can rapidly coselect for MRSA isolates carrying *fusC* within a staphylococcal cassette chromosome *mec* element (SCC*mec*), even in the absence of direct β-lactam exposure. This provides a plausible molecular basis for the abrupt and dramatic emergence of FA-resistant MRSA in NZ following the widespread use of topical FA (3). Further, we have clearly demonstrated that in this ST1 lineage, and under the experimental conditions tested, FA exposure can result in the rapid *in vitro* coselection of S. aureus clones that have genetically discrete and unlinked resistance mechanisms. The ability of FA to coselect for these clones is most likely linked to the high segregational stability of plasmid pNZAK1, which carries multiple unrelated antimicrobial resistance genes and appears to be effectively maintained by S. aureus even in the absence of direct selection.

Similarly, we observed that mupirocin exposure rapidly coselected for MDR isolates that carried pNZAK1, and which in this S. aureus lineage were also resistant to FA, from within a population of non-MDR strains. In NZ, previously high rates of mupirocin use led to an observed resistance rate in S. aureus of 28% in a 1999 survey (3). Interestingly, although topical FA had only recently been approved for clinical use at that time, the S. aureus FA resistance rate in 1999 was still 17% (3). Our observation that mupirocin coselects for FA-resistant strains in this lineage provides a biological explanation for this historically high rate of FA resistance, particularly in the context of our Bayesian modeling suggesting that *fusC* has been present in NZ ST1 isolates since the mid-1950s. Moreover, the ability of mupirocin to coselect for other resistance mechanisms colocated on mobile genetic elements highlights the need for regular surveillance of resistance in S. aureus in those settings where mupirocin is widely used (e.g., in universal decolonization regimens for the prevention of health care-associated infections). Our finding that *mupA* was present on a stably maintained plasmid (pNZAK1) in NZ ST1 isolates may also explain the ongoing high rate (8.3%) of mupirocin resistance in S. aureus in NZ (18). Derivatives of the pMW2 plasmid, including pNZAK1, are prevalent among community S. aureus isolates around the world (19). This observation therefore raises the possibility that the pMW2 family of plasmids might be important in the coselection of antimicrobial resistance and therefore the emergence of MDR community S. aureus strains. Of further clinical concern is the colocation of the *qacA* gene on pNZAK1; future work should explore the potential for mupirocin to coselect for strains displaying phenotypic tolerance to the biocide chlorhexidine.

It is well recognized that the injudicious use of systemic antibiotics has fueled the global rise of MDR bacteria. Here, by integrating comparative genomics and phenotypic analyses, we provide evidence that commonly used topical antibiotics may also play an underappreciated role in the emergence of antimicrobial resistance in S. aureus.

## MATERIALS AND METHODS

### Setting, bacterial isolates, and susceptibility testing.

NZ is an island nation in the South West Pacific, with a population of approximately 4.47 million. Between 18 and 20 March 2014, a national survey of antimicrobial resistance in S. aureus was undertaken, in which diagnostic microbiology laboratories in NZ were asked to refer all clinical (i.e., nonscreening) S. aureus isolates to the Institute of Environmental Science and Research (ESR), Wellington, New Zealand, for further analysis (18). MICs for FA and mupirocin were determined by agar dilution and interpreted using European Committee on Antimicrobial Susceptibility Testing (EUCAST) breakpoints (http://www.eucast.org/clinical_breakpoints/). *fusC*, *mupA*, and *qacA* were detected by PCR using previously described primers and methods (20, 21). For the purposes of this study, MRSA isolates that were phenotypically resistant to FA, mupirocin, and oxacillin were defined as multidrug resistant (MDR). All ST1 isolates obtained in the national survey were analyzed as part of this study.

### Whole-genome sequencing.

All ST1 isolates obtained from the national survey underwent whole-genome sequencing (WGS). DNA libraries were prepared using the Nextera XT DNA preparation kit (Illumina), and 2 × 150-bp sequencing was performed on the NextSeq platform (Illumina), as previously described (22). In addition, a representative ST1 isolate from NZ (NZ14487) underwent sequencing on the Pacific Biosciences RSII platform.

### Genomic, phylogenetic, and phylodynamic analyses.

The complete genome assembly of S. aureus NZ14487 was obtained using the SMRT Analysis system version 2.3.0.140936 (Pacific Biosciences). Raw sequence data were *de novo* assembled using the HGAP3 protocol, with a minimum seed read length of 5,000 base pairs (bp), genome size of 3 Mb, target coverage of 10, and overlapper error rate of 0.04. Polished contigs were further error corrected using Quiver version 1. The final assembly was checked using BridgeMapper version 1 in the SMRT Analysis system, and the consensus sequence was corrected with short-read Illumina data using Snippy version 2.5 (https://github.com/tseemann/snippy). The final sequences were annotated using Prokka version 1.11 (23).

Illumina sequence reads were *de novo* assembled using SPAdes version 3.6.1 (24) and annotated using Prokka version 1.11 (23). The program contig-puller (https://github.com/kwongj/contig-puller) was then used to identify, extract, and annotate assembled contigs containing *fusC*, *mupA*, and *qacA* (based on GenBank accession numbers WP_001033157, ABY70707, and CAB94808.1, respectively).

To characterize the population structure of ST1 S. aureus in NZ, the NZ ST1 collection was supplemented with sequence data of ST1 isolates from previously published studies and from a collection of ST1 isolates obtained in Australia through previous studies (25, 26). Further information for each isolate is available in Table S1 in the supplemental material.

Using Snippy version 2.5 (http://github.com/tseemann/snippy), the sequence reads of all ST1 S. aureus strains were mapped to, and variants called against, the closed reference genome NZ14487 generated during this study. Recombination detection was performed using Gubbins version 1.7 (27), and Bayesian phylogenomic and phylodynamic analyses were performed using BEAST version 1.8.3 (28). The details of these analyses are provided in the supplemental methods.

### Plasmid segregational stability assays.

Triplicate cultures of NZ14487 were grown overnight in brain heart infusion (BHI) broth supplemented with 100 mg/liter mupirocin. Each culture was diluted to a 0.5 McFarland standard (McF) in BHI broth before being diluted 1:100 into nonselective BHI broth or BHI broth containing 100 mg/liter mupirocin, performed in triplicate. Each culture was then grown for 24 h. In the same way, 5 serial passages of each culture were performed in either nonselective broth or broth containing 100 mg/liter mupirocin. At each time point, 100-μl aliquots were removed from each culture, serially diluted in phosphate-buffered saline (PBS), and spread onto nonselective BHI agar plates, which were incubated overnight. The next day, 100 colonies from each culture were cross-patched onto nonselective BHI agar and BHI agar containing 100 mg/liter mupirocin. Segregational stability was then calculated based on the percentage of colonies that were susceptible to mupirocin compared to the number of colonies that had grown on nonselective agar. Whole-genome sequencing was performed on representative isolates to confirm the identity of the isolates cultured and in susceptible isolates to confirm that plasmid loss had occurred.

### Competitive index assays.

Competitive index experiments were performed using a 1:1 ratio of test strains. The competing strains (MRSA meaning FA, mupirocin, and oxacillin resistant; and MSSA meaning FA, mupirocin, and oxacillin susceptible) were DMG1700937 (MRSA) versus DMG1700958 (MSSA), DMG1700944 (MRSA) versus DMG1701014 (MSSA), and DMG1700977 (MRSA) versus DMG1701019 (MSSA). The FA and mupirocin MICs for each strain can be found in Table S1. Cultures were grown overnight and then diluted to a 0.5 McFarland standard (McF) in BHI broth. Each pair of strains was then diluted 1:100 into 10-ml nonselective BHI broths or BHI broths containing 100 mg/liter mupirocin, 6 mg/liter oxacillin, or 8 mg/liter fusidic acid. Immediately after inoculation of the broths (time zero), 100-μl aliquots were removed from each assay culture, serially diluted in PBS, and spread onto nonselective BHI agar plates. These plates were incubated overnight, and 100 colonies from each culture were selected and cross-patched onto nonselective BHI agar and BHI agar containing either 6 mg/liter oxacillin, 8 mg/liter FA, or 100 mg/liter mupirocin. This process was repeated after 2 days and 7 days of incubation. The ratio of the competing input strains at each time point was determined based on the resistance profile of the selected colonies. WGS was then performed on representative isolates to confirm the identity of the strains growing in each patch.

### Accession number(s).

Sequence data for all NZ study isolates are available at the National Center for Biotechnology Information (NCBI) under accession number PRJNA412108.

## Supplementary Material

Supplemental material
