# Supplementary material for: Topical Antibiotic Use Coselects for the Carriage of Mobile Genetic Elements Conferring Resistance to Unrelated Antimicrobials in Staphylococcus aureus
Source: Antimicrob Agents Chemother. 2018 Jan 25;62(2):e02000-17. doi: 10.1128/AAC.02000-17 (PMC5786761; doi:10.1128/AAC.02000-17)
Supplement: Supplemental material [file supp_62_2_e02000-17__index.html]

Supplemental material 

# Topical Antibiotic Use Coselects for the Carriage of Mobile Genetic Elements Conferring Resistance to Unrelated Antimicrobials in Staphylococcus aureus

## Supplemental material

- Supplemental file 1 -

  Table S1

  XLSX, 20K
- Supplemental file 2 -

  Text S1

  PDF, 309K
