# Supplementary material for: Topical Antibiotic Use Coselects for the Carriage of Mobile Genetic Elements Conferring Resistance to Unrelated Antimicrobials in Staphylococcus aureus
Source: Antimicrob Agents Chemother. 2018 Jan 25;62(2):e02000-17. doi: 10.1128/AAC.02000-17 (PMC5786761; doi:10.1128/AAC.02000-17)
Supplement: Supplemental material [file AAC.02000-17_zac002186908s2.pdf]

SUPPLEMENTARY METHODS

Phylogenetic analysis and recombination detection

Reads for the 179 ST1 isolates were mapped against the 2,846,702 bp chromosome of the ST1 NZ14487 reference isolate. Prior to mapping, low-quality read ends (Phred score <30) and adapter sequences were removed using Trimmomatic (1). Mapping and subsequent single nucleotide polymorphism (SNP) calls were performed using Snippy v3.1 (https://github.com/tseemann/snippy), which uses BWA Mem (2) to map the reads to the reference and then calls the SNPs with FreeBayes (3). SNPs were called if the minimum number of reads covering the position was 10 and the minimum proportion of those reads which differ from the reference was 0.9. The whole genome alignment output from Snippy was used for downstream phylogenomic analysis.

A Maximum Likelihoood (ML) tree was inferred from the whole genome alignment using RAxML v8.2.9 (4). A total of 29,406 sites in the alignment were completely undetermined (i.e., containing only 'N' and '-') so were removed from further analysis. RAxML was run using the GTRCAT model, no rate heterogeneity, no ascertainment bias, rapid hill climbing, a seed of 6786987623 and empirical base frequencies. TempEst v1.5 (5) was used to examine the molecular clock-likeness of the ML tree. Using a best-fit root, the r^2 for root-to-tip branch length on time was 0.003 and the correlation coefficient was 0.053, indicating that the tree was not clock-like. There were three spikes in SNP density in the alignment. Analysis with PHASTER (6, 7) showed the SNP-dense regions to correspond with the positions of prophage sequences in the ARGS14487 reference strain (PHAGE_Staphy_phiJB_NC_028669 at positions 854197-927828, PHAGE_Staphy_phi2958PVL_NC_011344 at position 1545940-1608821 and PHAGE_Staphy_phiNM3_NC_008617at position 2069820-2117850).

The observation of mobile genetic elements and a weak molecular clock signal prompted us to search for and mask putative recombinant nucleotides in the alignment. This was performed using Gubbins v2.1.0 (8) with a RAxML tree builder, a GTRCAT site model and no site heterogeneity. Recombinant

blocks were detected as those regions containing at least three SNPs in alignment windows of between 100 and 10,000 sites. After four iterations, Gubbins reached a stable tree topology and so the analysis was stopped. As expected, Gubbins masked the three SNP-dense prophage regions as being among the recombinant regions detected by this analysis. Examination of the final post-Gubbins tree using TempEst with a best-fitting root showed a correlation coefficient of 0.497 and an r^2 of 0.247.

Phylodynamic analysis

From the 14,350-site filtered chromosomal SNP alignment, we performed a time-calibrated phylodynamic analysis using BEAST v1.8.3 (9). The best-fit model of nucleotide substitution was selected as GTR with no site heterogeneity after comparing 24 substitution models under the Akaike Information Criterion (AIC; 10) using MrModeltest (11) in PAUP 4.0b10 (12). We used empirical rather than estimated base frequencies as the SNPs were from a whole genome alignment with no ascertainment bias. To reconstruct the substitution rate in the ST1 lineage over time, we calibrated the temporal dimension of the analysis with tip dates specified as years of sample collection. We explored three different priors on the clock rate: strict, relaxed uncorrelated log-normal (UCLN; 13) and random local clock (14). Each prior used a continuous time Markov chain [CTMC] rate reference with an initial value of 1.0 (after 15). To estimate the temporal and spatial pattern of acquisition of plasmid-borne resistance determinants, we reconstructed discrete ancestral states over the phylogeny for country of collection and presence or absence of *blaZ*, *fusC*, *mecA*, *mupA* and *qacA* genes. Separate partitions were created for each trait and substitutions between states within each trait were modelled symmetrically. Changes in ST1 population size over time were estimated with a Bayesian Skyline reconstruction that used a piecewise-constant Bayesian Skyline coalescent prior on the tree (16, 17) with 45 groups (i.e., approximately one quarter of the number of isolates in the analysis). For each of the clock models, we performed 10 runs, with each run continuing for 50×10^6 cycles and samples taken every 1000 cycles. BEAST run-log files were compared using Tracer v1.5 (18). The random local clock (RLC) was deemed a poor fit for the ST1 dataset as none of the 10 runs converged on the same likelihood or rootHeight and each run had a very low effective sample size (ESS < 200) for most of the parameters of interest. For the relaxed clock, nine of the ten runs converged on the same likelihood

maximum after a burnin of 50% of the chain length and the ESSs for all parameters of interest were high. For the strict clock, all of the ten runs converged on the same likelihood maximum and ESSs were high after a burnin of 20% of the chain length. Bayes Factor (BF) analysis with 10,000 bootstrap replicates was used to compare the likelihoods of the relaxed and strict clocks. BF analysis showed that support for the strict clock over the relaxed clock is 'very strong' (see 19). That the coefficient of variation of the relaxed model was close to zero (0.202), rate variation over the tree was shown to be clock-like. The ten strict clock post-burnin log files were combined, resampling every 100,000th state. ESSs for all parameters of the combined run logs were >>200 (min. 459, max. 4890). Tree files for the strict clock were combined using the above parameters, and population size over time was reconstructed using the Bayesian Skyline plot tool in Tracer. The Maximum Clade Credibility (MCC) tree was annotated with posterior probabilities and common ancestor heights using the 4890 trees in the posterior sample.

REFERENCES

1. Bolger AM, Lohse M, Usadel B. 2014. Trimmomatic: a flexible trimmer for Illumina sequence data. Bioinformatics 30:2114-20.
2. Li H, Durbin R. 2009. Fast and accurate short read alignment with Burrows-Wheeler transform. Bioinformatics 25:1754-60.
3. Garrison E, Marth G. 2012. Haplotype-based variant detection from short-read sequencing. arXiv preprint arXiv:12073907.
4. Stamatakis A. 2015. Using RAxML to Infer Phylogenies. Curr Protoc Bioinformatics 51:6 14 1-14.
5. Rambaut A, Lam TT, Max Carvalho L, Pybus OG. 2016. Exploring the temporal structure of heterochronous sequences using TempEst (formerly Path-O-Gen). Virus Evolution 2.
6. Zhou Y, Liang Y, Lynch KH, Dennis JJ, Wishart DS. 2011. PHAST: a fast phage search tool. Nucleic Acids Res 39:W347-52.
7. Arndt D, Grant JR, Marcu A, Sajed T, Pon A, Liang Y, Wishart DS. 2016. PHASTER: a better, faster version of the PHAST phage search tool. Nucleic Acids Res 44:W16-21.
8. Croucher NJ, Page AJ, Connor TR, Delaney AJ, Keane JA, Bentley SD, Parkhill J, Harris SR. 2015. Rapid phylogenetic analysis of large samples of recombinant bacterial whole genome sequences using Gubbins. Nucleic Acids Res 43:e15.
9. Drummond AJ, Suchard MA, Xie D, Rambaut A. 2012. Bayesian phylogenetics with BEAUti and the BEAST 1.7. Molecular biology and evolution 29:1969-1973.
10. Akaike H. 1974. A new look at the statistical model identification. . IEEE Transactions on Automatic Control 19:716-723.

11. Nylander JAA. 2004. MrModeltest v2.2. Program distributed by the author, Uppsala, Sweden.
12. Swofford DL. 2002. PAUP*: Phylogenetic Analysis Using Parsimony (and other methods). v4.0b10. Sinauer Associates, Sunderland, MA.
13. Drummond AJ, Ho SY, Phillips MJ, Rambaut A. 2006. Relaxed phylogenetics and dating with confidence. PLoS Biol 4:e88.
14. Drummond AJ, Suchard MA. 2010. Bayesian random local clocks, or one rate to rule them all. BMC Biol 8:114.
15. Ferreira MAR, Suchard MA. 2008. Bayesian analysis of elapsed times in continuous-time Markov chains. Canadian Journal of Statistics-Revue Canadienne De Statistique 36:355-368.
16. Drummond AJ, Rambaut A, Shapiro B, Pybus OG. 2005. Bayesian coalescent inference of past population dynamics from molecular sequences. Mol Biol Evol 22:1185-92.
17. Drummond AJ, Nicholls GK, Rodrigo AG, Solomon W. 2002. Estimating mutation parameters, population history and genealogy simultaneously from temporally spaced sequence data. Genetics 161:1307-20.
18. Rambaut A, Suchard MA, Xie D, Drummond AJ. 2014. Tracer v1.5, Available from http://beast.bio.ed.ac.uk/Tracer,
19. Kass RE, Raftery AE. 1995. Bayes Factors. Journal of the American Statistical Association 90:773-795.
